# Assessment of Cardiovascular Risk and Arterial Stiffness in Patients With Human Immunodeficiency Virus

**DOI:** 10.7759/cureus.41784

**Published:** 2023-07-12

**Authors:** Mariana Freitas, Clarisse Neves, Helena Sarmento, Pedro Cunha, Jorge Cotter

**Affiliations:** 1 Nephrology, Centro Hospitalar de Trás-os-Montes e Alto Douro, Vila Real, PRT; 2 School of Medicine, University of Minho, Braga, PRT; 3 Internal Medicine, Hospital Senhora da Oliveira Guimarães, Guimarães, PRT

**Keywords:** haart, pulse wave velocity, hiv, cardiovascular risk, arterial stiffness

## Abstract

Background: Several studies suggest that patients infected with the human immunodeficiency virus (HIV) under highly active antiretroviral therapy (HAART) have a higher cardiovascular risk than the general population. Arterial stiffness is an independent predictor of cardiovascular events and can be measured through carotid-femoral pulse wave velocity (PWV). The objectives of this study were to characterize a sample of HIV-infected patients under HAART regarding cardiovascular risk, compare PWV values of this group with those of uninfected controls, and investigate predictors of PWV in the HIV-infected group.

Methods: PWV was measured, and data was collected from a sample of 125 HIV-infected patients under HAART. PWV measurements in the study group were compared with those in a control group of 250 subjects similar in sex, age, prevalence of hypertension, and type 2 diabetes mellitus (DM). A linear regression model was constructed to identify predictors of PWV in the HIV-infected group.

Results: In the HIV-infected group, composed mostly of men, the mean age and respective standard deviation were 48.6 ± 11.6 years. In this group, 112 individuals (89.6%) presented moderate to very high cardiovascular risk. Significant differences were found in median PWV between HIV-infected and control groups (8.56 vs. 8.00 m/s, *p* = .002). Age, peripheral systolic blood pressure, presence of DM, amount of alcohol consumed, and current CD4^+ ^T cell count were independent predictors of PWV in the HIV-infected group.

Conclusions: The HIV-infected group showed higher cardiovascular risk and arterial stiffness measurements than the general population. PWV may be an important predictor of subclinical cardiovascular disease in HIV-infected patients.

## Introduction

Human Immunodeficiency Virus (HIV) belongs to the family of human retroviruses (Retroviridae) and remains a serious public health problem. Around the world, in 2016, about 36.7 million people were living with HIV infection, with about 1.8 million new cases per year since 2010 [1].

The natural history of HIV infection has changed in the past few years. Since the introduction of highly active antiretroviral therapy (HAART), mortality has been considerably lower, and the life expectancy of HIV-infected patients has improved, approaching that described for the general population [2]. As a consequence of a lower mortality rate, morbidity due to chronic diseases has time to emerge [3].

Several studies suggest that people living with HIV infection have a higher cardiovascular risk than the general population [4-10]. In a cohort study with a group of HIV-infected patients and a control group of uninfected individuals from the same health system, Marcus et al. compared the incidence of ischemic stroke between groups and concluded that HIV-infected patients had a 40% increase in the risk of suffering an ischemic stroke, even when groups were adjusted for traditional cardiovascular risk factors [6]. The increase in cardiovascular risk in infected patients appears to have multiple causes, some of which are not completely clarified. Some causes pointed out in the literature are the direct action of the virus on vascular function, the chronic immune and inflammatory state caused by the virus, and the side effects of antiretroviral therapies [7]. It should be noted that it is necessary to take into account the therapeutic regimen used, as each group of drugs seems to present a distinct cardiovascular risk. Protease inhibitors appear to be those with the highest cardiovascular risk [8].

Taking into account that cardiovascular diseases are an important cause of morbidity and mortality and considering the increased cardiovascular risk presented by HIV-infected individuals, the advent of predictive markers of cardiovascular events is crucial. These predictive markers can alert the doctor and the patient early, enabling a preventive intervention.

Arterial stiffness is an independent predictor of cardiovascular events, and its study can provide important information regarding the individual’s cardiovascular morbidity or mortality risk [11]. The aortic pulse wave velocity, or carotid-femoral pulse wave velocity (PWV), is considered the gold standard for arterial stiffness assessment [11].

Arterial elastic properties are used for cardiovascular risk stratification in several populations, and the European Society of Cardiology (ESC) and the European Society of Hypertension (ESH) guidelines for the management of arterial hypertension in 2018 refer to PWV as a tool for asymptomatic hypertension-mediated organ damage assessment [12].

In a meta-analysis, Vlachopoulos et al. demonstrated that arterial stiffness, expressed as PWV, is a strong predictor of future cardiovascular events and all causes of mortality [13]. In the same meta-analysis, these researchers verified that for every 1m/s increase in PWV, the risk of developing a cardiovascular event increases by 14%. They also demonstrated that the ability to predict arterial stiffness is greater in individuals with a higher baseline cardiovascular risk [13].

Several studies have been developed to find out whether HIV-infected patients under HAART have higher arterial stiffness values than the general population, and the results have been controversial [4,7,14-15].

If we consider the change in the natural history of HIV infection under HAART, the increase in morbidity and the growing need to assess the cardiovascular risk of these patients since traditional cardiovascular risk calculators do not take into account the infection itself, the insertion in clinical practice of a marker that can predict cardiovascular events is an emerging need.

The present study aimed to characterize the cardiovascular risk of a sample of HIV-infected patients under HAART, compare the PWV values of this infected group with those of non-infected controls, and investigate predictors of PWV in the group of HIV-infected patients.

## Materials and methods

Selection of the study group

The selection of the study group was made through a convenience sample of patients who attended a medical appointment in Internal Medicine-Infectious Diseases at Senhora da Oliveira Hospital, Guimarães (SOHG) between July and October 2017 and who agreed to participate in this study. Inclusion criteria: diagnosis of HIV infection confirmed by Western blot; age ≥18 years; and being on a stable combination of HAART for ≥3 months, with ≥6 months of total cumulative exposure duration. A total of 150 patients who were interested in participating in the study were submitted to a brief interview, informed of the need to consult their clinical file, and submitted to non-invasive procedures for the determination of central blood pressure (CBP) and PWV. Those who met any of the following criteria were excluded: acute inflammatory disease or condition; chronic inflammatory conditions other than HIV, such as current neoplasia, autoimmune diseases, inflammatory bowel disease, or hepatitis C and B; the presence of usual medication that causes immunosuppression, such as chemotherapy or systemic corticosteroids; pregnancy or breastfeeding; and unsatisfactory values of the evaluated hemodynamic parameters. The final study group included 125 individuals infected with HIV.

Clinical interview, evaluation of hemodynamic parameters, and data collection

In the Center for the Research and Treatment of Arterial Hypertension and Cardiovascular Risk, Guimarães conducted a brief clinical interview, and the patients were questioned about personal habits and lifestyles, relevant personal and family history, and usual medication. Subsequently, peripheral blood pressure (PBP) was assessed according to the 2018 ESH/ESC guidelines [12] using an automatic device, the Omron HEM-711AC (Omron Healthcare, Inc., Bannockburn, Illinois). Subsequently, CBP and PWV were evaluated by the main investigator using the SphygmoCor system (Atcor Medical Pty Ltd., West Ryde, New South Wales, Australia). PWV was determined according to the recommendations and assumptions proposed by Van Bortel et al. [11]. Initially, two PWV assessments were performed; if they differed by less than 0.5m/s, the average of the values was calculated; when the two assessments differed by more than 0.5m/s, a third assessment was performed, considering as the final result the median of these values. It should be noted that only measurements with a standard deviation of <10% were considered for this study. The reference values considered for the analysis of PWV were those proposed by Boutouyrie et al. [16]. Regarding CBP, it was determined by the non-invasive method of tonometry of radial artery applanation, according to the recommendations proposed by McEniery et al. [17]. Data regarding demographic and anthropometric characteristics, relevant medical history, and laboratory parameters were collected from physical files in the Center for the Research and Treatment of Arterial Hypertension and Cardiovascular Risk and also from digital files using the *SClinic* software.

Cardiovascular risk assessment

The 10-year absolute cardiovascular risk of the study group was calculated using the SCORE (Systematic Coronary Risk Evaluation) tool, according to the orientation of the General Health Direction “Cardiovascular Risk Assessment SCORE (Systematic Coronary Risk Evaluation)”, updated in 2015 [18]. The 10-year risk of developing cardiovascular disease (CVD) was also calculated using the Framingham Score (FS) using an automatic calculator, “General CVD Risk Prediction Using Lipids,” available on the Framingham Heart Study website [19].

Control group

The control group emerged from a database owned by the Department of Internal Medicine of the SOHG. Gender, age, hypertension, and diabetes mellitus (DM) are variables identified in the literature as determinants of PWV. For this reason, a descriptive analysis of the study group (HIV-infected patients) regarding these variables was performed, and a matched control group was required and made available by the person in charge of the Center for the Research and Treatment of Arterial Hypertension and Cardiovascular Risk in a proportion of two controls to one infected.

Statistical analysis

Statistical analysis was performed using the IBM Corp. Released 2016. IBM SPSS Statistics for Windows, Version 24.0. Armonk, NY: IBM Corp. Categorical variables were described through absolute frequencies and respective percentages; continuous variables were presented as mean and standard deviation (SD) if they followed a normal distribution or median and interquartile range (IQ) if they were not normally distributed. Normality was tested using the Shapiro-Wilk test or the analysis of asymmetry and kurtosis, according to the assumptions described by Kim [20]. For all tests, a 95% confidence interval was established, so all tests with a probability value (p) <0.05 were considered statistically significant. To compare groups in terms of categorical variables, we used the chi-square test or Fisher’s exact test (if the percentage of cells in the table with an expected frequency of less than 5 was greater than 20%); regarding continuous variables, the Mann-Whitney test was used. To study the correlation between the continuous variable PWV and the remaining variables, the point-biserial correlation (rpb) was used for the bicategorical variables, the Pearson correlation coefficient (r) for the normally distributed continuous variables, and the Spearman correlation (rs) in the case of continuous variables not normally distributed or ordinal categorical variables. The effect size of the correlations was considered small, medium, or large when there were values close to .10, .30, or .50, respectively. Finally, a multiple linear regression model was developed to understand which variables in the HIV-infected group are implicated in the increase in PWV (dependent variable).

Ethical considerations

This project was submitted and approved by the Subcommittee of Ethics for Life and Health Sciences of the University of Minho and by the Committee of Ethics for the Health of SOHG. All patients signed an informed consent form to participate in the study.

## Results

Characterization of the HIV-infected patients’ group

The HIV-infected group included 125 patients with ages ranging from 23 to 84 years, with a median (IQ) age of 48 (15) years. The male gender was the most prevalent (79.2%). It was found that 65 individuals (52.0%) had a body mass index (BMI) that corresponds to overweight, with 14 (11.2%) being obese. The complete characterization of the HIV-infected group regarding demographic, anthropometric, clinical, and laboratory variables is summarized in Table 1.

**Table 1:** Clinical characteristics according to the study group *Statistically significant p-value (< .05); ** We considered arterial target organ damage when PWV > 10 m/s; ***Reference values from the publication by Boutouyrie, et al. [16]; HIV: human immunodeficiency virus; BMI: body mass index; IV: intravenous; CVD: cardiovascular disease; CKD: chronic kidney disease; FPG: fasting plasma glucose; TC: total cholesterol; cHDL: high-density lipoprotein cholesterol; cLDL: low-density lipoprotein cholesterol; PSBP: peripheral systolic blood pressure; PDBP: peripheral diastolic blood pressure; CSBP: central systolic blood pressure; CDBP: central diastolic blood pressure; PWV: pulse wave velocity; TOD: arterial target organ damage.

	HIV-infected group N = 125	Control group N = 250	p-value
Age, years [Median (IQ)]	48 (15)	47 (14)	p = .62
Male gender [n (%)]	99 (79.20)	196 (78.40)	p = .86
Black race [n (%)]	4 (3.20)		
BMI, Kg/m^2 ^[Median (IQ)]	25.0 (5.70)	26.5 (5.10)	p < .001*
Current smoking [n (%)]	60 (48.00)	121 (48.40)	p = .94
Contact with IV drugs [n (%)]	28 (22.40)		
Contact with inhaled drugs [n (%)]	32 (25.60)		
Current drug consumption [n (%)]	8 (6.40)		
Current alcohol consumption [n (%)]	52 (41.60)		
Alcohol, g/day [Median (IQ)]	<0.001 (20.0)		
Sedentary lifestyle [n (%)]	31 (24.80)		
Arterial hypertension [n (%)]	28 (22.40)	55 (22.00)	p = .93
Diabetes Mellitus [n (%)]	17 (13.60)	28 (11.20)	p = .50
Dyslipidemia [n (%)]	70 (56.00)	203 (81.20)	p < .001*
Established CVD [n (%)]	10 (8.00)	3 (1.20)	p = .001*
Family history of early CVD [n (%)]	16 (12.80)		
CKD [n (%)]	5 (4.00)	15 (6.00)	p = .42
FPG, mg/dL [Median (IQ)]	97 (15)	82 (17)	p < .001*
TC, mg/dL [Median (IQ)]	163 (48)	198 (50)	p < .001*
cHDL, mg/dL [Median (IQ)]	44 (15)	48 (15)	p = .002*
cLDL, mg/dL [Median (IQ)]	89 (39)	123 (43)	p < .001*
Triglycerides, mg/dL [Median (IQ)]	112 (91)	102 (79)	p = .021*
Creatinine, mg/dL [Median (IQ)]	0.94 (0.26)	0.80 (0.19)	p < .001*
PSBP, mmHg [Median (IQ)]	129 (19)	128 (17)	p = .873
PDBP, mmHg [Median (IQ)]	76 (13)	79 (13)	p = .105
CSBP, mmHg [Median (IQ)]	117 (20.0)		
CDBP, mmHg [Median (IQ)]	77.0 (13.00)		
PWV, m/s [Median (IQ)]	8.56 (2.10)	8.00 (2.20)	p = .002*
TOD ** [n (%)]	23 (18.40)	38 (15.20)	p = .43
PWV > 2SD of mean*** [n (%)]	9 (7.20)	19 (7.6)	p = .54

In our study group of HIV-infected patients, the age at diagnosis of HIV infection was, on mean ± SD, 38.0 ± 11.6 years. The evolution time of the HIV infection in the study group was 10.7 ± 5.97 years. Regarding laboratory parameters associated with HIV infection, it was found that the CD4+ T cell count at diagnosis, nadir, and current have medians (IQ) of 260 (335), 189 (210), and 598 (497) cells/mm3, respectively. More information about the parameters related to HIV infection is shown in Table 2.

**Table 2:** Characterization of HIV infection-related variables in HIV-infected groups * The HIV viral load detection threshold considered was 40 copies/mL; HIV: human immunodeficiency virus; AIDS: acquired immunodeficiency syndrome.

HIV infection related variable	
Age at diagnosis, years (N=125) [Mean ± SD]	38.0 ± 11.6
HIV infection evolution time, years (N=125) [Mean ± SD]	10.7 ± 5.97
CD4^+ ^T cell count at diagnosis, cells/mm^3^ (n=120) [Median (IQ)]	260 (335)
CD4^+^ T cell nadir count, cells/mm^3^ (n=122) [Median (IQ)]	189 (210)
Current CD4^+^ T cell count, cells/mm^3^ (N=125) [Median (IQ)]	598 (497)
Current CD4^+^/CD8^+ ^Ratio, % (N=125) [Median (IQ)]	0.69 (0.67)
HIV1 viral load at diagnosis, copies/mL (n=119) [Median (IQ)]	96622 (404300)
Maximum HIV1 viral load, copies/mL (n=120) [Median (IQ)]	163708 (430379)
Current HIV1 viral load detectable*, n (%) (N=125)	15 (12.0)
AIDS stage (N=125) [n (%)]	46 (36.8)

The duration of exposure to any HAART scheme has a mean ± SD of 8.26 ± 4.97 years. The mean exposure time to nucleoside reverse transcriptase inhibitors (NRTI) was 8.15 ± 5.03 years. The cumulative exposure time to abacavir ranged between 0 and 13.6 years in our group of HIV-infected patients. We verified a median (IQ) cumulative exposure time to non-nucleoside reverse transcriptase inhibitors (NNRTI) of 3.50 (8.34) years. The cumulative exposure time to efavirenz had a median (IQ) of 0.08 (6.00) years, with 10 patients (8.00%) currently undergoing a therapeutic regimen that integrates it. Regarding protease inhibitor (PI) agents, we verified that 58 individuals (46.4%) were exposed at some point to HAART regimens containing a drug from this group. Currently, 25 participants (20.0%) are exposed to a therapeutic scheme containing a PI. A more detailed analysis of the HAART-related variables in the group of patients infected with HIV can be found in Table 3.

**Table 3:** Characterization of HAART associated variables in the HIV-infected group *More than 50% of the patients have never been exposed to PI; HAART: highly active antiretroviral therapy; HIV: human immunodeficiency virus; NRTI: nucleoside reverse transcriptase inhibitor; NNRTI: non-nucleoside reverse transcriptase inhibitor; PI: protease inhibitor; II: integrase inhibitor.

HAART associated variable	N=125
HAART, years of cumulative exposure (Mean ± SD)	8.26 ± 4.97
NRTI, years of cumulative exposure (Mean ± SD)	8.15 ± 5.03
NNRTI, years of cumulative exposure [Median (IQ)]	3.50 (8.34)
PI, years of cumulative exposure* [Median (IQ)]	0 (5.17)
II, years of cumulative exposure* [Median (IQ)]	0 (0.88)
Tenofovir, years of cumulative exposure (Mean ± SD)	4.44 ± 2.59
Abacavir, years of cumulative exposure* [Median (IQ)]	0 (0)
Efavirenz, years of cumulative exposure [Median (IQ)]	0.080 (6.00)
Darunavir, years of cumulative exposure* [Median (IQ)]	0 (0)
Lopinavir, years of cumulative exposure* [Median (IQ)]	0 (0.25)
Ritonavir, years of cumulative exposure* [Median (IQ)]	0 (3.75)
Dolutegravir, years of cumulative exposure * [Median (IQ)]	0 (0)
Current exposure to PI [n (%)]	25 (20.0)
Current exposure to tenofovir [n (%)]	106 (84.8)
Current exposure to efavirenz [n (%)]	10 (8.00)

According to the SCORE tool, most of the HIV-infected patients had a moderate cardiovascular risk, corresponding to a total of 77 patients (61.6%), and only 13 individuals (10.4%) had a low cardiovascular risk. The remaining results related to the cardiovascular risk assessment of the HIV-infected group are presented in Table 4.

**Table 4:** Characterization of the cardiovascular risk in a group of HIV-infected patients *Calculated according to the orientation of the General Health Direction “Cardiovascular Risk Assessment SCORE (Systematic Coronary Risk Evaluation)”, updated in 2015 [18]; **10-year risk to develop a cardiovascular disease, calculated using an automatic calculator “General CVD Risk Prediction Using Lipids” available on the Framingham Heart Study website, applicable only between 30 and 74 years old [19].

Cardiovascular risk assessment tool	
SCORE (N=125)*	
Low cardiovascular risk, n (%)	13 (10.4)
Moderate cardiovascular risk, n (%)	77 (61.6)
High cardiovascular risk, n (%)	12 (9.60)
Very high cardiovascular risk, n (%)	23 (18.4)
Framingham Score (n=114)** [Median (IQ)]	8.80 (11.6)
Vascular Age (n=114)** [Median (IQ)]	53.0 (20.0)

Comparison between HIV-infected and control group

In our study, we compared the HIV-infected group, composed of 125 infected individuals, with a control group of 250 individuals without HIV infection. The control group was similar to the HIV-infected group in terms of age, gender, hypertension, and diabetes mellitus prevalence. No difference was found in the peripheral systolic blood pressure (PSBP) or peripheral diastolic blood pressure (PDBP) values between groups. Regarding analytical parameters, it was observed that the HIV-infected group presented total cholesterol (TC), high-density lipoprotein cholesterol (cHDL), and low-density lipoprotein cholesterol (cLDL) significantly lower than the control group. On the other hand, the control group showed significantly higher levels of fasting plasma glucose (FPG), triglycerides, and creatinine. There was no association between the presence (or absence) of HIV infection and gender, current smoking, hypertension, DM, or chronic kidney disease (CKD) variables. However, the presence of dyslipidemia and established cardiovascular disease showed a significant association with the presence (or absence) of HIV infection. Dyslipidemia was less frequent in the infected group (56.0%) than in the control group (81.2%), and established CVD was more frequent in the infected group (8.00%) than in the control group (1.20%). Finally, significant differences in PWV values were found, with the HIV-infected group having a significantly higher median PWV (median = 8.56 m/s; IQ = 2.10) than the control group (median = 8.00m/s; IQ = 2.20). A more detailed view of the characteristics of both groups can be found in Table 1.

Correlation between PWV and other study variables in the HIV-infected group

In our results, age was associated with an increased PWV, and it was the variable that showed the greatest effect size on PWV. Other variables that presented a positive correlation with PWV were the amount of alcohol ingested per day, sedentary lifestyle, hypertension, DM, dyslipidemia, and peripheral and central blood pressure. No correlations were found between PWV and the presence of established CVD or CKD in the HIV-infected group. Current smoking and current consumption of drugs of any kind were habits that showed a negative correlation with PWV, with a small effect size. Regarding HIV-infection-associated variables, we found that the age at diagnosis of the HIV infection exhibited a positive correlation with PWV, with a large effect size. There was also a significant positive correlation between PWV and viral loads at diagnosis and maximum. CD4+ T cell counts at diagnosis, minimum, and current were negatively correlated with PWV. Concerning variables related to HAART, only the cumulative exposure time to HAART, current use of PI, cumulative exposure time to PI, and cumulative exposure time to darunavir was positively correlated with PWV, all with a small effect size. Dolutegravir was the only drug that showed a negative correlation with PWV, with a small effect size (data not shown). Lastly, both scores used to assess cardiovascular risk were positively correlated with PWV, with a large effect size. Table 5 summarizes the data related to the study's correlations, as well as the respective correlation coefficients.

**Table 5:** Correlation between PWV and other study variables in the HIV-infected group * Statistically significant p-value (< .05); PWV: pulse wave velocity; HIV: human immunodeficiency virus; BMI: body mass index; IV: intravenous; CVD: cardiovascular disease; CKD: chronic kidney disease; FPG: fasting plasma glucose; TC: total cholesterol; cHDL: high-density lipoprotein cholesterol; cLDL: low-density lipoprotein cholesterol; PSBP: peripheral systolic blood pressure; PDBP: peripheral diastolic blood pressure; CSBP: central systolic blood pressure; CDBP: central diastolic blood pressure; PWV: pulse wave velocity; AIDS: acquired immunodeficiency syndrome; HAART: highly active antiretroviral therapy; NRTI: nucleoside reverse transcriptase inhibitor; NNRTI: non-nucleoside reverse transcriptase inhibitor; PI: protease inhibitor; II: integrase inhibitor.

Variable	p-value	Correlation coefficient
Age	< .001>	r (125) = .59
Gender	.83	r_pb_ (125) = .019
Race	.26	r_pb_ (125) = .10
Abdominal girth	.017*	r (125) = .21
BMI	.29	r (125) = .095
Current smoking	.010*	r_pb_ (125) = -.23
Contact with IV drugs	.42	r_pb_ (125) = -.074
Contact with inhaled drugs	.14	r_pb_ (125) = -.13
Current drug consumption	.043*	r_pb_ (125) = -.18
Current alcohol consumption	.27	r_pb_ (125) = .10
Amount of alcohol per day	.004*	r_s_ (125) = .26
Sedentary lifestyle	.004*	r_pb_ (125) = .26
Hypertension	< .001>	r_pb_ (125) = .39
Dyslipidemia	.010*	r_pb_ (125) = .23
Diabetes Mellitus	< .001>	r_pb_ (125) = .38
Established CVD	.094	r_pb_ (125) = .15
Family history of early CVD	.84	r_pb_ (125) = -.018
CKD	.053	r_pb_ (125) = .17
FPJ	.001*	r_s_ (125) = .29
TC	.003*	r (125) = .27
cHDL	.12	r_s_ (125) = .14
cLDL	.093	r (125) = .15
Triglycerides	.057	r_s_ (125) = .17
Creatinine	.61	r_s_ (125) = .046
PSBP	< .001>	r_s_ (125) = .42
PDBP	.009*	r_s_ (125) = .23
CSBP	< .001>	r_s_ (125) = .47
CDBP	.019*	r_s_ (125) = .21
Age at diagnosis of HIV infection	< .001>	r (125) = .53
HIV infection evolution time	.13	r (125) = .14
CD4^+^ T cell count at diagnosis	< .001>	r_s_ (120) = -.37
CD4^+^ T cell nadir count	< .001>	r_s_ (122) = -.32
Current CD4^+^ T cell count	.018*	r_s_ (125) = -.21
Current CD4^+^/CD8^+^ ratio	.26	r_s_ (125) = -.10
HIV1 viral load at diagnosis	.018*	r_s_ (119) = .22
Maximum HIV1 viral load	.034*	r_s_ (120) = .19
Current HIV1 viral load detectable	.39	r_pb_(125) = .078
AIDS stage	.91	r_pb_ (125) = -.011
HAART, years of cumulative exposure	.038*	r (125) = .19
NRTI, years of cumulative exposure	.073	r (125) = .16
NNRTI, years of cumulative exposure	.63	r_s_ (125) = .043
IP, years of cumulative exposure	.026*	r_s_ (125) = .20
Current exposure to PI	.027*	r_pb_ (125)= .20
II, years of cumulative exposure	.28	r_s_ (125) = -.097
Abacavir, years of cumulative exposure	.18	r_s_ (125) = -.12
Tenofovir, years of cumulative exposure	.24	r (125) = .11
Current exposure to tenofovir	.29	r_pb_(125) = -.095
Efavirenz, years of cumulative exposure	.36	r_s_ (125) = .083
Current exposure to efavirenz	.21	r_pb_ (125) = .11
Lopinavir, years of cumulative exposure	.26	r_s_ (125) = .10
Ritonavir, years of cumulative exposure	.055	r_s_ (125) = .17
Darunavir, years of cumulative exposure	.011*	r_s_ (125) = .23
SCORE	< .001>	r_pb_(125) = .48
Framingham Score	< .001>	r_s_ (114) = .50
Vascular Age	< .001>	r_s_ (114) = .56

Multiple linear regression model

A linear regression model was constructed to identify predictors of PWV in the group of HIV-infected patients. In this model, independent variables were tested as those that showed the highest correlations in the previously described correlation tests, as well as those that were most pointed out in the literature. In Table 6, we presented the multiple linear regression model that we found to predict the variance of PWV [F (5, 119) = 30.0, p <.001, R2 = .56]. It was found that age, PSBP, presence of DM, amount of alcohol ingested per day, and current CD4+ T cell count independently and significantly explain 56.0% of the variance of the PWV in the HIV-infected group.

**Table 6:** Multiple linear regression model for variables associated with PWV (dependent variable) PWV: pulse wave velocity; PSBP: peripheral systolic blood pressure.

	B	Standard error	Beta	t	95% confidence interval for B	p-value
Constant	1.96	0.84		2.35	[0.31; 3.62]	.020
Age	0.059	0.008	0.48	7.28	[0.043; 0.075]	< .001>
PSBP	0.030	0.005	0.35	5.54	[0.019; 0.041]	< .001>
Diabetes Mellitus	0.72	0.27	0.17	2.61	[0.17; 1.26]	.010
Amount of alcohol per day	0.006	0.003	0.13	2.01	[<0.001; 0.012]	.046
Current CD4^+ ^T cell count	-0.001	<0.001	-0.13	-2.14	[-0.001; <0.001]	.035

## Discussion

The sample of HIV-infected patients studied in our work had a prevalence of traditional cardiovascular risk factors similar to those described for other populations of infected individuals found in the literature. The only exceptions were age, prevalence of DM, and current smoking, which appear to be more elevated in our sample of infected patients [15,21,22].

Regarding cardiovascular risk assessment, it was found that 28% of HIV-infected individuals presented a high or very high cardiovascular risk. However, it was not possible to compare these values since no studies were found using the same tool. On the other hand, through the Framingham Score, we find out that the median risk of developing a CVD in 10 years in our group of HIV-infected individuals was 8.80% (IQ = 11.6). This value is higher than that found in the literature [22], which allows us to suppose that the group of HIV-infected individuals under HAART followed at SOHG presents a higher cardiovascular risk than the general population. These results agree with the literature [6,9,10,21-24].

The increased risk of atherosclerotic cardiovascular events in individuals living with HIV infection seems to have multiple causes, including the senescence associated with HIV infection itself, chronic inflammation and immune activation caused by the virus, as well as metabolic disorders related to HAART [23]. Since arterial stiffness is a powerful predictor of cardiovascular mortality and PWV is the gold standard for its determination [11], one of the aims of this study was to evaluate the role of inflammation and immune activity caused by HIV under HAART in arterial stiffness through the assessment of PWV. Significant differences were found between the PWV values of HIV-infected and control groups [median (IQ): 8.56 (2.10) vs. 8.00 (2.20) m/s, p = .002). These results agree with previous studies [7,25] but are contrary to some others [4,15]. The higher mean age of the HIV-infected individuals in our study may partly explain the opposition to the results of the referred studies, which evaluated younger populations. A strength of the present study is the size of the control group. It should be noted that our control group presented significantly higher values for BMI, TC, and cLDL and a higher prevalence of dyslipidemia; nevertheless, it was the infected group that had the highest median for PWV. As mentioned above, the remaining main determinants of arterial stiffness were controlled, and this seems to validate the assumption that, independently of traditional cardiovascular risk factors, HIV and respective HAART increases arterial stiffness. In a cohort study with a representative sample of the population of Guimarães and Vizela, Cunha et al. concluded that the referred population had PWV values higher than expected for a low cardiovascular-risk area such as Portugal [26]. The results of these authors in some way validate our finding because, even compared to a control group originating from a population with increased arterial stiffness (higher than expected for a Portuguese population), the PWV values of the HIV-infected patients proved to be significantly superior.

Concerning the PWV-associated factors in our study group of HIV-infected patients, a strong positive association was found between age and PWV. This result is consistent with the literature [15,16]. Previous studies have verified an association between male gender and PWV in HIV-infected populations [4,15]. The same did not occur in the present study, which can be explained by the low proportion of females. Monteiro et al. did not find an association between PWV and BMI [15], with results similar to ours.

A negative association was found between PWV and current smoking, but smokers were younger and therefore had lower PWV values, which may have been an important confounding factor. The non-association between smoking habits and PWV is reported in the bibliography [4,15]. According to Eckard et al. [4], current alcohol consumption (any amount vs. no consumption) is related to an increased PWV in HIV-infected patients [median (first quartile, third quartile): 6.0 (5.6, 6.5) m/s vs. 5.4 (4.8, 6.0) m/s; p = .0002]. The authors did not evaluate the amount of alcohol consumed, and that was one of the limitations pointed out by them in their own study [4]. In our study, we found a positive association between the amount of alcohol consumed and the PWV value. Alcohol consumption disorders are described among the HIV-infected population [27], which gives more importance to these results.

In their studies, Boutouyrie et al. pointed out age and blood pressure as highly conditioning factors for PWV [16]. According to these authors, treated or untreated DM, treated hypertension, and dyslipidemia are other conditions that predict PWV [16]. These results are consistent with those found in our study.

We did not verify a correlation between PWV and the presence of established CVD; nevertheless, correlations with strong effect sizes were found between PWV and cardiovascular risk assessment tools (SCORE and Framingham Score). These results seem to validate the use of PWV as a measure of arterial stiffness in the prediction of cardiovascular events in HIV-infected individuals, as reported in previous studies for the general population [11].

Regarding the HIV-associated variables, it was found that older ages at diagnosis were associated with higher PWV values. Age itself seems to be a confounding factor in this correlation since no correlation was found between PWV and HIV infection evolution time, which is consistent with the consulted literature [4,15].

In our findings, PWV presented a negative association with the CD4+ T cell nadir count, a result that has been previously described in the literature [28]. The current CD4+ T cell count also demonstrated a negative association with the PWV, an association already described [4,7].

A recent meta-analysis compared groups of HIV-infected individuals on and without HAART, and the results demonstrated that HIV-infected individuals under HAART presented the highest PWV values, especially when exposed to PI [8]. The antiretroviral drugs most often associated with an increased cardiovascular risk in the literature are abacavir, efavirenz, and PI [8,29,30]. In the present study, cumulative exposure time to PI, current use of PI, and cumulative exposure time to darunavir were associated with higher PWV values. There was no correlation between PWV and abacavir or efavirenz; however, the exposure time of the study population to these drugs was reduced.

In the multiple linear regression model that we found for the group of HIV-infected patients under HAART, only the variables age, PSBP, presence of DM, amount of alcohol ingested per day, and current CD4+ T cell count proved to be independent predictors of PWV. This model explains 56.0% of the PWV variance. It was not surprising to find age, PSBP, and the presence of DM in this model. These are, indeed, variables already widely described as predictors of PWV [16]. Eckard et al., in their study with a population of HIV-infected patients, had already described alcohol consumption as an independent predictor for PWV in a model that explained 21.1% of its variance [4]. In our model, the amount of alcohol consumed was considered, which is an advantage. Furthermore, our model shows that the current immunity status of these patients influences the PWV.

This study presents the limitations associated with a cross-sectional study; in other words, it does not allow the establishment of cause-effect relationships. We used a convenience sample of HIV-infected patients who went to the medical appointment, which may have induced a selection bias since patients with worse control may not have been approached. In our study, no distinction was made between treated and untreated dyslipidemia and hypertension, which can affect PWV [16]. Despite the successful matching of the study groups regarding age, gender, and presence of hypertension or DM, the same was not possible for other relevant variables, such as dyslipidemia, given the high prevalence of dyslipidemia in the population from which the control group came. Lastly, this study does not allow us to conclude whether it is HIV itself or HAART that provides the increase in cardiovascular risk since all individuals were on HAART.

## Conclusions

In conclusion, the present study demonstrated that the group of HIV-infected individuals followed in SOGH has a higher cardiovascular risk than the general population, as well as higher arterial stiffness values. The strong association between PWV and cardiovascular risk scores seems to validate the use of PWV as a predictor of subclinical cardiovascular disease in this group of patients. In the group of HIV-infected patients, PWV was significantly and independently affected by age, PSBP, presence of DM, amount of alcohol consumed, and current CD4+ T cell count. The predictors found for PWV in the group of HIV-infected patients may be important because, except for age, all of them could be modifiable. Since traditional risk calculators do not consider the patient's immune status, PWV seems to be a more complete marker of subclinical cardiovascular disease. Hereafter, it is recommended to perform longitudinal studies with larger and multicenter samples. Case-control studies with patients grouped according to the HAART scheme may also be interesting to improve the associations between arterial stiffness and HAART.
